# Isolation and genome sequence annotation of KillerTomato, a newly discovered cluster EE bacteriophage infecting *Microbacterium*

**DOI:** 10.1128/MRA.00852-23

**Published:** 2023-10-25

**Authors:** Omer Almahie, Breanna Danklefsen, R. Scott Graves, Sariah M. Hepworth, Rylee T. Mathison, Emily A. Sullivan, Paige R. Wood, Skylar Bartholomew, Reganne Brewster, Dylan J. Hunt, Carlos Serna, Jack F. Shurley, Anna S. Grinath, Michael A. Thomas

**Affiliations:** 1 Department of Biological Sciences, Idaho State University, Pocatello, Idaho, USA; DOE Joint Genome Institute, Berkeley, California, USA

**Keywords:** bacteriophage, student-directed research, SEA-PHAGES

## Abstract

Discovered in Pocatello, Idaho, soil near a tomato garden, siphovirus KillerTomato infects *Microbacterium foliorum* NRRL B-24224. KillerTomato is a lytic cluster EE phage with a 17,442-bp genome and 68.6% GC content. Of 25 genes, 20 were assigned putative functions, including a putative tail assembly chaperone protein with a programmed frameshift and an endolysin.

## ANNOUNCEMENT

The discovery and annotation of bacteriophages are an important step in the development of phage therapy strategies for fighting bacterial infections (1). Here, we describe the characteristics of a lytic bacteriophage that infects *Microbacterium foliorum*.

**Fig 1:**
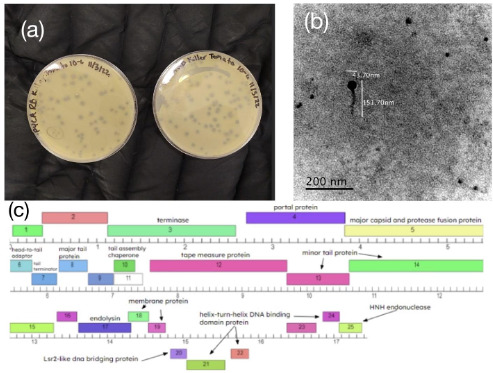
Characterization of bacteriophage KillerTomato. (**a**) KillerTomato forms round, medium-sized plaques. (**b**) Siphovirus morphology of KillerTomato with a capsid diameter of approximately 44 nm and a tail length of approximately 108 nm. Image produced by a Zeiss EM900 TEM with an accelerating voltage of 80 kV and uranyl acetate negative staining; 40–50 virions were measured. (**c**) The genome of KillerTomato contains 25 putative protein-coding genes (portrayed by the boxes with numbers depicting the gene number) on both forward and reverse strands.

KillerTomato was found in garden soil on 1 September 2022 on the Idaho State University campus in Pocatello, Idaho (42.867222°N, 112.428889° W), following established procedures (2, 3). Soil samples were washed with PYCa liquid media and filtered with a 0.22-μm filter. The filtrate was inoculated with *Microbacterium foliorum* NRRL B-24224. After incubation with shaking at 20°C for 48 h, the culture was filtered and the filtrate was plated on PYCa agar with M. foliorum and incubated at 20°C for 48 h. Plaques formed by KillerTomato were purified through three additional rounds of plating (Fig. 1a). Transmission electron micrographs of KillerTomato revealed a siphovirus with a long tail (Fig. 1b).

Using a Promega Wizard DNA clean up kit, KillerTomato DNA was extracted from a high-titer lysate and prepared for sequencing using a NEBNext Ultra II FS kit. DNA was sequenced using a shotgun sequencing approach on an Illumina MiSeq (v3 reagents). Sequencing generated 650,649 single-end 150-bp reads with 5,321-fold coverage. The raw reads were trimmed and assembled with Newbler v2.9 using default parameters, yielding a single contig; Consend v29 was used to check for completeness and accuracy and determine phage termini, both using default settings (4 – 6). KillerTomato has a genome length of 17,442 bp and a 3′ single-stranded sticky overhang of 9 bases (5′-CCCGCCCCA-3′). It has a GC content of 68.6%, which matches its host (68.7%) (7). KillerTomato belongs to cluster EE based on gene content and similarity to other EE phages in the Actinobacteriophage database (8).

The genome of KillerTomato was auto-annotated with PECAAN (http://discover.kbrinsgd.org) and DNAmaster v5.23.6 (http://cobamide2.bio.pitt.edu) with manual inspection and confirmation. Genemark v2.5 (9) and Glimmer v3.0 (10) were used to analyze the start sites and coding potential of each gene and for initial auto-annotation. We used Phamerator v454 (11) for cluster assignment and synteny analysis. Starterator (http://phages.wustl.edu/starterator/) was applied to compare start sites manually annotated for other EE phages to KillerTomato. HHPred v2.0 (12) (searches of pdb, pfamA, and NCBI CD databases) and BLAST v2.13.0 (13) (searches of the PhagesDB and NCBI nr databases) were used to predict putative gene functions. No tRNAs were identified using Aragorn v1.2.41 (14) and tRNA-SE v2.0 (15). Using default parameters, the annotation process yielded 25 protein-coding genes, 20 of which could be assigned functions (Fig. 1C).

Consistent with other EE phages, KillerTomato has a small genome with several fused genes (16). All but three genes (gp20 to gp22) are transcribed on one strand. KillerTomato has a putative tail assembly chaperone gene with a programmed frameshift (gp10 and gp11). KillerTomato also appears to encode an endolysin (gp17), a key protein involved in hydrolyzing the peptidoglycan layer of its host during a productive infection and that offers a promising strategy for treating infections by antibiotic-resistant bacteria (17). Along with plaque morphology, comparison with other cluster EE phages, which lack immunity repressor and integrase functions, suggests KillerTomato is a lytic phage.

## Data Availability

GenBank and SRA accession numbers for KillerTomato are OR159653 and SRX19690840, respectively.
